# Insights into physiological roles of unique metabolites released from *Plasmodium*-infected RBCs and their potential as clinical biomarkers for malaria

**DOI:** 10.1038/s41598-018-37816-9

**Published:** 2019-02-27

**Authors:** Divya Beri, Ghania Ramdani, Balu Balan, Darshak Gadara, Mukta Poojary, Laurence Momeux, Utpal Tatu, Gordon Langsley

**Affiliations:** 1 Department of Biochemistry, Indian Institute of Science, Bangalore, 560012 India; 2 Inserm U1016, Cnrs UMR8104, Cochin Institute, Paris, 75014 France; 3 Laboratoire de Biologie Cellulaire Comparative des Apicomplexes, Faculté de Médecine, Université Paris Descartes - Sorbonne Paris Cité, Paris, France

## Abstract

*Plasmodium* sp. are obligate intracellular parasites that derive most of their nutrients from their host meaning the metabolic circuitry of both are intricately linked. We employed untargeted, global mass spectrometry to identify metabolites present in the culture supernatants of *P*. *falciparum-*infected red blood cells synchronized at ring, trophozoite and schizont developmental stages. This revealed a temporal regulation in release of a distinct set of metabolites compared with supernatants of non-infected red blood cells. Of the distinct metabolites we identified pipecolic acid to be abundantly present in parasite lysate, infected red blood cells and infected culture supernatant. Further, we performed targeted metabolomics to quantify pipecolic acid concentrations in both the supernatants of red blood cells infected with *P*. *falciparum*, as well as in the plasma and infected RBCs of *P*. *berghei*-infected mice. Measurable and significant hyperpipecolatemia suggest that pipecolic acid has the potential to be a diagnostic marker for malaria.

## Introduction

*Plasmodium* sp are the etiological agent of malaria that causes >400,000 deaths in tropical countries around the world. Intracellular parasites have unique metabolic pathways and exceptional strategies that help them hijack their host’s metabolism for their own survival and propagation. In the intraerythrocytic stage of its life cycle, *Plasmodium* parasites actively replicate and confer massive metabolic demands on their hosts. Metabolic indicators of malaria include elevated oxidative stress, heightened host inflammatory response, hyperhomocystinemia, hemoglobinuria due to excessive catabolism of hemoglobin by growing parasites, hypoglycemia, anemia and recently observed hypoarginemia^1–4^. However, none of these modulations in metabolite levels are restricted exclusively to parasite infection. Diagnosis of malaria remains a major challenge and most of the metabolite markers are common with symptomatically similar diseases^5^. Thus, a metabolite that is specifically regulated during *P*. *falciparum* intra-erythrocyte growth could provide a sensitive technique for parasite detection.

A hallmark of malaria pathogenesis is the export of substances to cytosol of the infected red blood cell (iRBC) and surrounding medium/plasma^6^. Thus, metabolomics of infected red blood cells and/or culture supernatants/plasma can provide valuable insights into parasite metabolism and host-parasite interactions^7^. Metabolomics in malaria is a relatively new, but fast expanding field, and previous studies have indicated important parasite metabolic pathways^5,8–11^. For example, global profiling of *P*. *falciparum*-infected RBCs led to the observation that the parasite has chorismite and alpha linolenic pathways that are absent in its human host^8,10^. Plasma/urine analysis of infected patients has identified metabolite candidates associated with cerebral malaria and absent in mild malaria^5^. Also, attempts have been made to identify and map the biochemical pathways perturbed in the parasite when administered with drug candidates belonging to the Malaria Box^12^. Genomic analysis of *Plasmodium* sp revealed an absence of gluconeogenesis, *de novo* purine synthetic pathways and amino acid biosynthetic pathways^13^, and metabolic studies confirmed these predictions^2^.

It is well known that *Plasmodium*-infected RBCs are permeable to several metabolites, which otherwise can’t permeate through RBCs^6^. Global metabolic profiling of culture supernatants can therefore provide a snap shot of metabolites effluxed out of iRBC some of which act *in trans* on neighboring infected and non-infected cells^14–16^. It can also be applied to identifying metabolites unique to *Plasmodium* metabolism^16,17^. The current study was designed to profile metabolites released into the culture supernatants of synchronized *P*. *falciparum*-infected red blood cells at 8 h (predominance of ring stage), 24 h (predominance of trophozoite-stage) and 40 h (predominance of schizont-stage) post-invasion. We stopped the analysis at 40 h to avoid release of iRBC content concomitant with merozoite egress and iRBC rupture at 48 h. Metabolites detected in culture supernatants represent therefore, only a fraction of metabolites produced *P*. *falciparum-*infected RBCs. Nonetheless, at different times post-invasion significant changes were observed in both the amount and identity of individual metabolites released into the supernatant of *P*. *falciparum*-infected red blood cells. As levels of released pipecolic acid were the most significantly different targeted metabolomics was used to quantify pipecolic acid concentrations in both the supernatants of red blood cells infected with *P*. *falciparum*, as well as in the plasma of *P*. *berghei*-infected mice. Measurable and significant hyperpipecolatemia suggest that pipecolic acid has the potential to be a diagnostic marker for malaria.

## Results

### Global metabolic profiling of spent culture supernatant of *Plasmodium falciparum*-infected red blood cells

**Figure 1:**
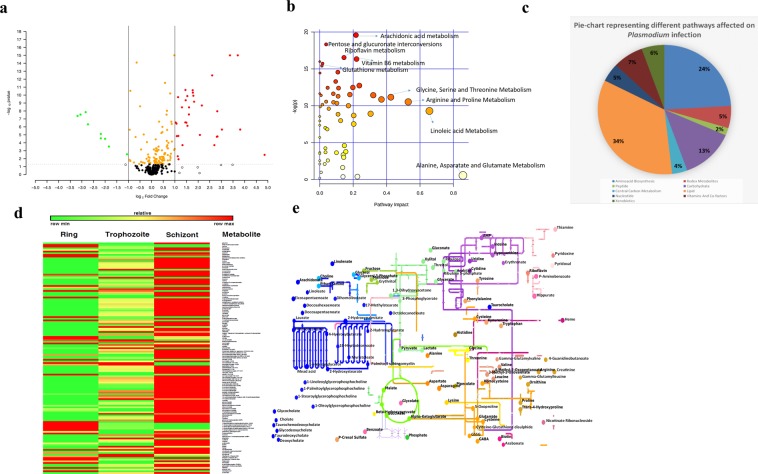
Global metabolic profiling of supernatants of *P*. *falciparum*-infected erythrocytes. (**a**) Volcano plot depicting fold change (X-axis) in 141 metabolites between normal RBCs and *P*. *falciparum*-infected erythrocytes (iRBCs) with their statistical significance indicated by the Y-axis. The scatter points on the left and right of zero in the fold change (FC) axis represent down-regulated and up-regulated metabolites, respectively. Circles towards the top in the Y-axis represent metabolites that have been affected with statistical significance (P- value < 0.05; separated by a grey line) while the circles towards the lower half of the Y-axis represent metabolites with P-value > 0.05. Each circle is representative of a metabolite. Red circles indicate metabolites with a FC > 2 and P-value < 0.05. Green circles indicate metabolites with a FC < 2 and P-value < 0.05. Yellow circles indicate metabolites that are affected with a P-value < 0.05, but not significant with respect to FC threshold. Colored black circles indicate metabolites that are statistically insignificant (P- value > 0.05) and have insignificant FC. Open black circles indicate metabolites that have a fold change < or >2, but are not statistically significant (P-value < 0.05). The data presented correspond to the statistical analysis of n = 5 independent biological replicates and 4 technical replicates. (**b**) Pathway impact analysis plot showing metabolic pathways affected across time and infection with statistical significance. The radius of the circle represents the number of metabolites identified in each pathway. The color gradient from white to red is representative of the cumulative statistical significance of the pathway and the individual pathway impact on the overall metabolic change observed. (**c**) Pie chart representing the percentage of metabolites involved in different processes that changed between the normal group and the infected group in *Plasmodium* culture supernatant. Most of the metabolites that were up/down regulated in the infected group belonged to the lipid (34%) and amino acid metabolic pathways (24%). Redox metabolites, xenobiotics and carbohydrate metabolism also contributed a significant percentage of metabolites that exhibited a change in the two groups. (**d**) Heat map representation of changes in metabolite levels measured in culture supernatants of *P*. *falciparum-*infected RBCs at ring, trophozoite and schizont stages. Green rectangles indicate a down regulation in the metabolite level and red rectangles indicate an increase in metabolite level. Most metabolites show an increase in their levels as *P*. *falciparum* intra-erythrocyte development progresses to the schizont stage. (**e**) The schematic representation of a global metabolic map showing the 141 metabolites identified from *P*. *falciparum-*infected RBC culture supernatants. Colours in the map represent the different pathways in which these metabolites are involved. In each of these pathways, the highlighted metabolites have been identified in the current study.

A total of 141 metabolites were measured at the different intraerythrocyte developmental stages (ring, trophozoite and schizont). To highlight the most affected metabolites, a volcano plot was constructed (Fig. 1a). Only nine metabolites showed lower metabolic levels in iRBC-supernatants compared to uninfected RBC-supernatants (green colour dots) with fold change (FC) of more than 2 and P-value < 0.05. A total of 41 metabolites were upregulated with a FC of more than 2 and P-value < 0.05. on *P*. *falciparum* infection (red coloured dots). To further analyze the impact each pathway had on the cumulative change in metabolite levels across time, we constructed a pathway impact analysis plot (Fig. 1b). This depicts the contribution and importance of each of the pathways displaying a change with infection and over the time course of intra-erythrocyte development. Amino acid metabolism and lipid metabolism dominate the plot with a significant contribution from glutathione and vitamin B2 and B6 metabolism. This reiterates the importance of these pathways in parasite metabolism. The 141 metabolites were assigned their respective metabolic pathways and lipid metabolism had the maximum number of detected metabolites (34%), followed by amino acid biosynthesis (24%) (Fig. 1c). We further constructed a heat map illustrating the levels of metabolites across the three-time points; 8 h, 24 h and 40 h corresponding to rings, trophozoites and schizonts, respectively (Fig. 1d). Most metabolites remained unchanged at the earliest time point but were significantly affected at later time points corresponding to trophozoite- and schizont-iRBC. Taking these metabolites, a metabolic map of *Plasmodium* using KEGG and PathwayProjector was constructed to illustrate the dynamic changes occurring in the different pathways during the RBC infection cycle. The schematic was drawn using Pathway Projector and KEGG^18,19^ and highlighted metabolites in the map are deregulated at different stages of the RBC infection cycle. These metabolites are involved in a number of pathways including carbohydrate, amino acid and lipid metabolism. The map provides a snapshot of the metabolic rewiring occurring during the course of *P*. *falciparum* erythrocyte infection (Fig. 1e).

### Profile of extracellular metabolites changes during *P*. *falciparum* intra-erythrocyte development

**Figure 2:**
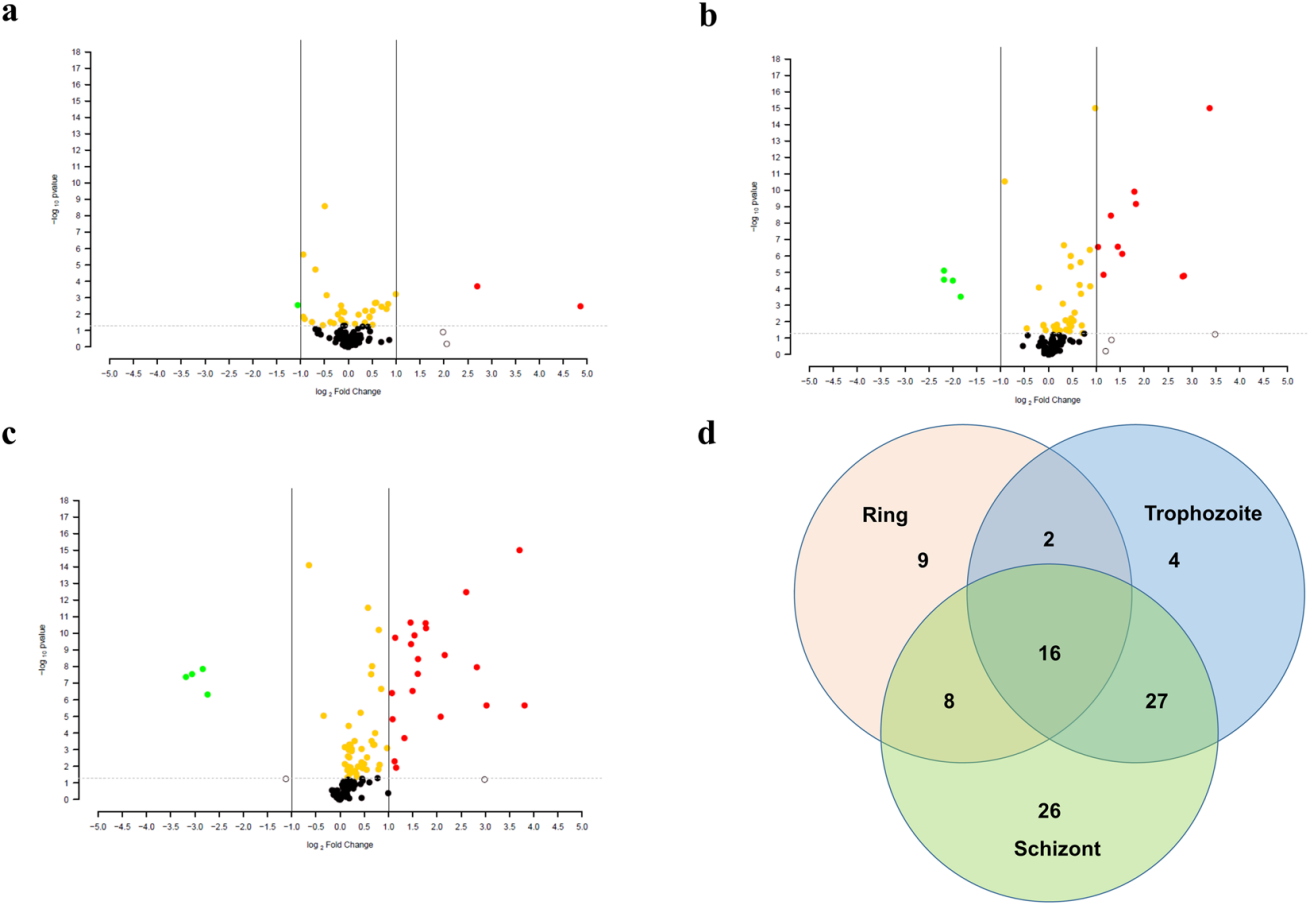
Metabolite profiling of different asexual stages of *P*. *falciparum*-infected RBCs. Volcano plot combing statistical significance and fold change observed in metabolites in the culture supernatant of *P*. *falciparum*-infected RBC compared to normal RBC at different time points of. Red circles indicate metabolites with a fold change >2 and statistical significance P < 0.05. Green circles indicate metabolites with a fold change <2 and statistical significance P < 0.05. Yellow circles indicate metabolites, which have statistical significance, but insignificant fold change values. Coloured black circles indicate metabolites that are statistically not significant and have insignificant fold change. Open black circles indicate metabolites that have a fold change <or >2, but are not statistically significant. (**a**) Volcano plot for affected metabolites between culture supernatant of uninfected RBCs and ring-infected RBCs. Only two metabolites show a significant upregulation and one metabolite shows a significant down regulation. (**b**) Volcano plot depicting affected metabolites between culture supernatant of uninfected RBCs and trophozoite-infected RBCs. A few metabolites show significant upregulation. (**c**) Volcano plot depicting metabolites affected between culture supernatant of uninfected RBCs and schizont-infected RBCs. At this stage, many metabolites show significant upregulation implying active change in the host metabolome following *P*. *falciparum*-infection of RBC. (**d**) Venn diagram representing a stage-specific metabolic correlation analysis, with each coloured circle representing a specific *P*. *falciparum* intra-erythrocyte developmental stage. It represents the metabolites (P < 0.05) that are affected in the supernatants of RBCs infected at the ring, trophozoite and schizont stages and their uniqueness/overlap between different life cycle stages.

To profile metabolites whose levels changed during intra-erythrocyte development volcano plots were constructed for rings (8 h), trophozoites (24 h) and schizonts (40 h). The majority of metabolites present in the culture supernatant of ring-infected RBC did not show any significant FC with only the levels of three metabolites altered, reflecting that the rings are the most metabolically inactive intra-erythrocyte stage of the parasite (Fig. 2a). Nine metabolites were significantly up regulated and 4 down regulated at the trophozoite stage with increased metabolites belonging to glycolysis and amino acid metabolism pathways (Fig. 2b). Metabolites displaying decreased levels belong to lipid metabolism pathways highlighting the dynamic nature of lipids during intra-erythrocyte development of *P*. *falciparum*^20^. At 40h schizont-iRBC stage, levels of 21 metabolites increased and most of these were be assigned to amino acid metabolism with a few to carbohydrate metabolism (Fig. 2c). Levels of 4 metabolites decreased and they all belong to lipid metabolism indicating the importance of this pathway in *P*. *falciparum*-infection of erythrocytes. Figure 2d summarizes (with P-value < 0.05) differentially altered (both up regulated and down regulated) metabolites in the culture supernatant of *P*. *falciparum*-infected compared to uninfected RBC.It highlights metabolites that are unique and common to the different intra-erythrocyte development stages of *P*. *falciparum*. Only 16 metabolites appeared common among all the three stages, schizonts and trophozoites shared 27 metabolites, while rings, trophozoites and schizonts had 9, 4 and 26 unique metabolites, respectively; illustrating that there are significant differences in metabolic pathways during intra-erythrocyte development of *P*. *falciparum*.

### Growth of *P*. *falciparum* alters extracellular redox milieu: Modification of cellular redox metabolism

**Figure 3:**
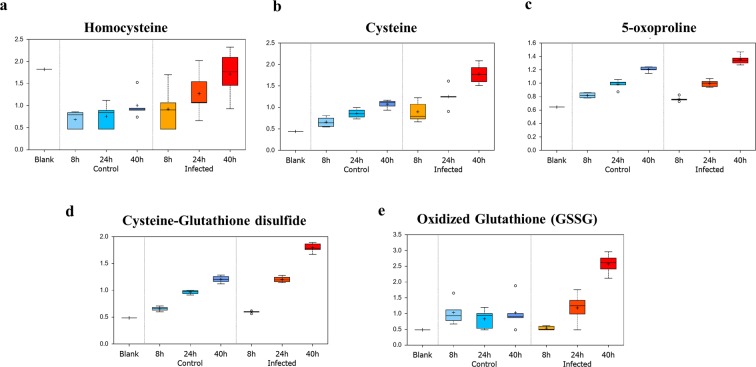
*P*. *falciparum* infection of RBC leads to efflux of redox metabolites in the culture supernatant. Box plot for change in level of detected redox metabolites at 8 h, 24 h and 40 h in control RBC vs infected RBC culture medium. Y-Axis of the box plots represents scaled intensity and X-Axis indicates the treatment group. (**a**) Homocysteine in the medium was ~2-fold elevated at 40 h in infected vs control (P = 0.05, n = 5). (**b**) The level of the amino acid cysteine was moderately upregulated in the infected RBC culture supernatant at 24 h and 40 h (P = 0.0002, n = 5; P = 0.0001, n = 5). (**c**) 5-oxoproline was mildly affected by *P*. *falciparum*-infection of RBC at 40 h (P = 0.0025, n = 5). (**d**) Cysteine-Glutathione disulfide was also slightly, but significantly, affected at 40 h (P < 0.0005, n = 5). (**e**) At 40 h oxidized glutathione was observed to be over 2-fold upregulated in the supernatant of *P*. *falciparum*-infected RBC compared to normal RBC supernatant (P = 0.0002, n = 5).

Glutathione serves as a powerful antioxidant to combat oxidative stress, which is particularly prevalent in RBCs due to their oxygen carrying capacity, lack of mitochondria, and inability to synthesize new proteins/enzymes. Infection with *P*. *falciparum* has been associated with increased release of metabolites related to glutathione synthesis and oxidative stress^1^. Figure 3 displays sustained accumulation of the glutathione precursors homocysteine and cysteine increasing with time post invasion. Oxidized glutathione (GSSG), the gamma-glutamyl cycle intermediate 5-oxoproline, and the biochemical marker of oxidative stress, cysteine-glutathione disulfide, exhibited reductions at the early time points (8 h), but became elevated 40h post-invasion (Supplementary Table 1). Furthermore, the gamma-glutamyl amino acids gamma-glutamylvaline and gamma-glutamylleucine were also elevated at later time points, suggestive of increased gamma-glutamyl cycle activity for recycling of glutathione with longer infection periods. Taken together, these findings indicate an initial redox response to *P*. *falciparum* infection that is sufficient to combat oxidative stress, but as the parasite develops it becomes overwhelmed and unable to maintain redox homeostasis. It is also important to note that increasing culture times alone contribute to elevations in oxidative stress in uninfected RBCs (control in Fig. 3).

### *In vitro* growth of *P. falciparum* modifies levels of metabolites belonging to major metabolic pathways

**Figure 4:**
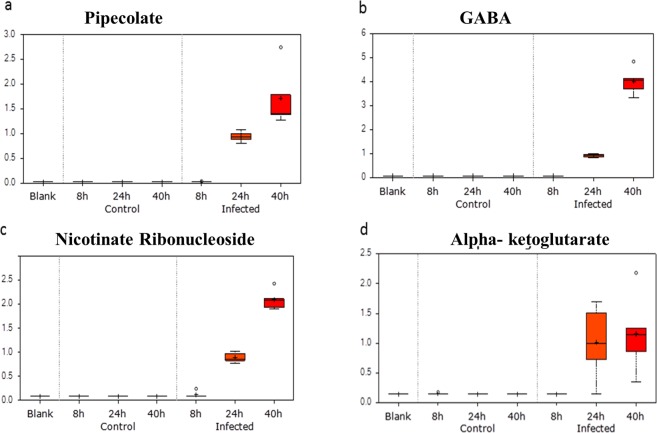
Probable metabolite markers of *P*. *falciparum*-infection of RBC. Box plot for change in levels of detected metabolites at 8 h, 24 h and 40 h in the spent medium of control RBC vs *P*. *falciparum*-infected RBC. Y-Axis of the box plots represents scaled intensity and X-Axis indicates the treatment group. (**a**) Plot shows absence of pipecolate in the normal group and a significant presence in the infected group, especially at later time points when parasites are in their late trophozoite/schizont stage (~30-fold upregulated at 24 h and ~60-fold upregulated at 40 h; P < 0.0005; n = 5). (**b**) Plot shows a complete absence of gamma aminobutyrate in the control RBC and in ring-infected RBC. It was elevated at 24 h (10-fold upregulated; P < 0.0005, n = 5) and 40 h (45-fold upregulated; P < 0.0005, n = 5) exclusively in the *P*. *falciparum*-infected RBC group. (**c**) Box plot shows absence of nicotinate ribonucleoside in the supernatants of control RBC and ring-infected RBC. It was significantly elevated in medium obtained from trophozoite-infected RBC (10-fold upregulated; P < 0.0005, n = 5) and schizont-infected RBC (25-fold upregulated; P < 0.0005, n = 5). (**d**) Box plot shows absence of alpha-ketoglutarate in non-infected RBC supernatants. It was exclusively present in the spent medium of the infected RBC at 24 h (~7-fold upregulated; P < 0.0005; n = 5) and 40 h (~8- fold; P = 0.0001; n = 5).

**Figure 5:**
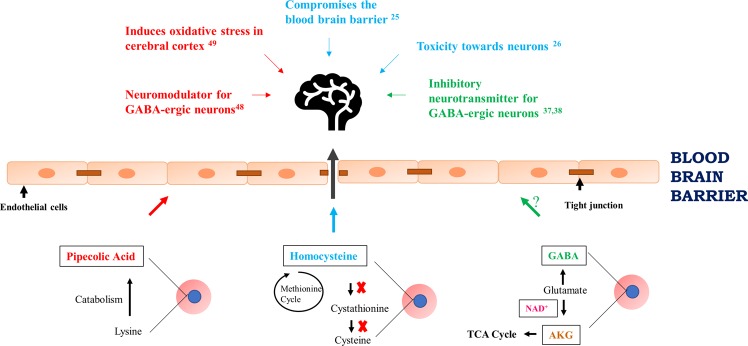
Probable effects of extracellular metabolites present in culture supernatant of *P*. *falciparum*-infected RBCs on cerebral malaria: The lower part of the schematic model summarizes the biochemical pathways and their interconnectivity operating in P. falciparum for the generation of metabolites: pipecolic acid, AKG, GABA, NAD^+^ and homocysteine. On release of these metabolites in the bloodstream, pipecolic acid (red arrow), GABA (green arrow) and homocysteine (cyan arrow) can possibly breach the blood brain barrier and consequently affect the neurons contributing to cerebral malaria pathogenicity. The known effects of these metabolites in the brain have been described with the above-mentioned color coding. Representative image of brain obtained from licensed version of Microsoft Office 365 Powerpoint® Product ID: 00202-51569-36211-AA678. AKG: alpha ketoglutarate; GABA: Gamma amino butyric acid; red circles and blue circles represent red blood cells and intra-erythrocytic stage of *Plasmodium falciparum*, respectively.
  Pipecolate is also a metabolite produced during amino acid metabolism being the catabolic product of lysine metabolism^27^. Earlier clinical studies have shown up regulation of pipecolate in *Plasmodium*-infected patients, as well as mice infected with *P*. *berghei*^11,28–31^. This metabolite is also seen to be elevated in pediatric cerebral malaria and has been shown to be specific for *Plasmodium*-induced fever and not in non-malarial fever. A significant change of 30-fold at 24 h and 60-fold at 40 h was observed in levels of pipecolic acid (Fig. 4a). Earlier studies have suggested that elevated pipecolic acid levels are due to changes in gut microbiota^11,29^. However, our study demonstrates that the up regulation of the metabolite is exclusively attributable to the parasite metabolism.
  3-Methyl-2-oxovaleric acid and 4-methyl-2-oxopentanoate are the alpha-keto analogue of branched chain amino acids (BCAA) isoleucine and leucine, respectively (KEGG Pathway map 00280)^18^. They arise from incomplete breakdown of BCAA and are known acidogens, neurotoxins and metabotoxins and at the 40 h schizont-iRBC stage both are ~3.5-fold up regulated (Supplementary Table 1). *P*. *falciparum* possesses a branched chain keto acid dehydrogenase (BCKDH), but lacks other enzymes of this pathway, which explains up regulation of these two metabolites^32^. Due to their neurotoxicity, they may also have important implications in cerebral malaria (Fig. 5).
(C) Carbohydrate metabolism: Extracellular metabolites typical of glycolysis and the tricarboxylic acid cycle (TCA) exhibited changes in their levels across the time points. RBCs lack mitochondria and therefore rely solely on glucose and glycolysis for production of ATP. Erythrocyte infection by *P*. *falciparum* increased levels of several metabolites associated with glucose utilization, including the glycolysis intermediates 3-phosphoglycerate, pyruvate and lactate with increases of 13-fold, 3-fold and 2–3-fold, respectively (Figs S2A, S3A). The most affected metabolite of the TCA cycle was alpha ketoglutarate (AKG) that apart from being a rate-determining intermediate in the TCA cycle also participates in transamination reactions linking carbohydrate to amino acid metabolism (Figs S2A, S3A). We observed a 7-fold (24 h) and 8-fold (40 h) increase in extracellular AKG compared to a complete absence of extracellular AKG from non-infected RBC (Figs S2A and S3A). In addition, the pentose phosphate pathway (PPP) is an important source of the cofactor NADPH that plays a role in anabolic processes as well as maintenance of the tripeptide antioxidant glutathione in its reduced state^33^. Similar to what we observed for glycolysis, *P*. *falciparum*-infection of RBCs also increased levels of the PPP intermediate ribulose 5-phosphate (17-fold induction at 40 h) and pentose sugars such as xylitol, indicative of increased activity of this alternative pathway (Fig. S3A). Greater release of metabolites related to glucose metabolism may be indicative of increased utilization through glycolysis and the PPP and/or a function of increased membrane permeability, as accumulation of metabolites occurred in a stepwise fashion with time post-invasion.
(D) Nucleotide Metabolism: Upon *P*. *falciparum* infection, nucleotides were the least affected class of metabolites. RBCs contain mM quantities of ATP that is released from erythrocytes by deformations, as they pass from arteries to vessels and capillaries and by many stimuli such as hypoxia^34^. As demonstrated previously ATP leached from RBC can bind to ATP receptors present on the RBC surface, or it can be rapidly converted to adenosine by the successive action of ecto-nucleotidases CD39 and CD73, where the resulting adenosine binds to its receptor ADORA2B^16^. Among the 141 metabolites, AMP was first detected 24 h post-infectionwith a large increase 40 h post-infection. AMP when converted to adenosine by the ecto-5′-nuclotidase CD73 bind to ADORA2B leading to an increase in intracellular levels of cAMP^35^. Previously we have demonstrated the *in vivo* relevence for extracellular AMP in *P*. *berghei* infected mice. When injected with an inhibitor of CD73 to block the conversion of AMP into adenosine, we had observed that inhibition of CD73 over a period of 10 days reduced parasite load by 60%^16^.

The 10 most up-regulated and down-regulated metabolites observed at each development stage are shown in Supplementary Figs (S1–S3) and detailed below:

(A) Lipid metabolism: As evident in Fig. 1c, 34% of metabolites can be assigned to lipid metabolism. Identified were lipid metabolites belonging to different classes: essential fatty acids, medium-chain fatty acids, long-chain fatty acids, branched fatty acids, lysophospholipids, sphingolipids, glyceroplipids, monoacylglycerol and lipids belonging to bile metabolism. The parasite is known to perform *de novo* fatty acid biosynthesis, as well as importation of fatty acids into the infected RBCs^10^. The most affected class was lysophosphatidylcholines (LPCs) that contains plasma lipids derived from phosphatidylcholines by the action of phospholipase A2, or oxidation. LPCs are believed to be important signaling molecules and are components of oxo-low density lipoproteins^20^. Chain lengths of LPC have been reported shortened in *Plasmodium*-infected patients^21^.
  Infection of RBCs with *P*. *falciparum* is associated with pronounced changes in cellular membrane rigidity, deformity, and adhesiveness^22,23^ and the levels of several metabolites related to membrane dynamics were found significantly altered in supernatants of *P*. *falciparum*-infected RBCs. Specifically, pronounced accumulation of membrane phospholipid precursors/degradation products such as choline, ethanolamine, and glycerol 3-phosphate along with significant reductions in choline-containing lysolipids could be observed (Figs S1–3, and Supplementary Table 1). Changes in these metabolites is consistent with membrane remodeling by the parasite and increased levels of trans-4-hydroxyproline (a derivative of proline), certain essential and long-chain fatty acids, erythronate [formed when the glycosylation moiety N-Acetyl-D-glucosamine (GlcNAc) is oxidized], and sphingolipid palmitoyl sphingomyelin were also observed extracellularly. Taken together, alterations in these metabolite levels highlight the biochemical changes underlying *P*. *falciparum*-induced alterations in membrane dynamics.
(B) Amino acid metabolism: The levels of many metabolites present in the culture supernatant belonging to amino acid metabolism were higher in the supernatants of *P*. *falciparum*-infected erythrocytes. Notably, gamma amino butyrate (GABA) and pipecolate are significantly elevated at the trophozoite and schizont stage (Fig. 4a,b). *P*. *falciparum* parasites can convert alphaketoglutarate (Fig. 4c) to glutamate that can be further converted to GABA. There is an operational glutamate/GABA transaminase (PF3D7_060880) in the mitochondria and cytoplasm, but unlike its close relative *T*. *gondii*, the parasite lacks the complete GABA shunt pathway and cannot catabolize GABA to succinyl-coA, which may explain its higher level in the infected medium^24^. The complete absence of GABA in the uninfected controls indicates that the metabolite is parasite-specific and can be a prospective metabolic marker for *P*. *falciparum*. Furthermore, secreted GABA by *P*. *falciparum* schizont-infected erythrocytes could have important implications, as it could bind to GABA-ergic receptors expressed by neurons. In this context, high levels of extracellular homocysteine (Fig. 3a) have been implicated in neurological damage and disrupting the blood brain barrier^25,26^. The extrusion of GABA and homocysteine by *P*. *falciparum* schizont-infected erythrocytes could have important clinical implications in understanding coma associated with cerebral malaria (Fig. 5).

### Identification of potential biomarkers of P. falciparum infection

We analyzed metabolites released from *P*. *falciparum-*iRBC and this not only provided valuable insights into the metabolic pathways of the pathogen, but also provided an opportunity to develop unique biomarkers for malaria. Detection of such metabolites in *P*. *falciparum*-infected-human serum could serve as a sensitive and accurate diagnostic tool for malaria. Four extracellular metabolites were very different between infected and uninfected erythrocytes with pipecolic acid being the most prominent (Fig. 4a). Pipecolic acid is an important intermediate in lysine degradation known to be elevated in peroxisomal disorders like Zellweger’s syndrome and Refsum’s disease^36^. Hyperpipecolatemia in humans is associated with neurological symptoms and kidney and liver dysfunctions; all of which are hallmarks associated with severe *P*. *falciparum* infections. The second highly abundant metabolite in *Plasmodium*-infected culture supernatant was GABA that’s derived from glutamate and is an inhibitory neurotransmitter (Fig. 4b). There are contrary reports on permeability of the blood brain barrier to this metabolite; however, during cerebral malaria when the blood brain barrier is compromised, GABA may play a role by acting on GABA-ergic neurons^37,38^. To our knowledge, the only other clinical diseases that result in elevation of plasma GABA concentration are hepatic encephalopathy and mood-related disorders, where interestingly pipecolic acid plays a regulatory role^39,40^. The third metabolite present in the infeceted culture supernatant exclusively was Nicotinate Adenine Dinucleotide (NAD) (Fig. 4c). NAD^+^, together with phosphorylated NAD^+^ (NADP^+^) and reduced NAD^+^ (NADH, NADPH), serves as important redox cofactors and enzyme substrates. It is a critical cofactor involved in many enzymatic reactions. NAD is the cofactor for two important metabolic processes in the parasite: glycolysis and glutamate dehydrogenase-mediated nitrogen assimilation or dissimilation. While glycolysis is the central energy producing metabolic pathway in the parasite, the latter is important for assimilation of ammonia in the form of glutamate, or dissimilation of ammonia to alphaketoglutarate to be fed into the TCA cycle^41^. *P*. *falciparum* is known to be completely dependent on exogenous source of nicotinamide/nicotinic acid^13,42^ and although *de novo* NAD synthesis is absent in *P*. *falciparum*, a salvage pathway exists that is known to be critical for parasite metabolism^43^. The spent culture medium of parasite-infected RBC exhibited 10-fold and 25-fold higher levels at 24 h and 40 h, respectively compared to spent medium of non-infected RBCs (Fig. 4c). Previous studies have demonstrated up regulation of NAD in iRBC and consequent depletion of nictotinamide, a precursor of NAD, in the iRBC spent medium^2,42^. Increased serum/plasma levels of in NAD have not been reported as implicated in any other disease condition and thus, may serve as a unique biomarker for malaria. The fourth metabolite uniquely present in culture supernatants from *P*. *falciparum* infected-red blood cells was alphaketoglutarate (AKG) that is an important TCA cycle intermediate found 7-fold (24 h) and 8-fold (40 h) more abundant (Fig. 4d). Interestingly, all these metabolites are interlinked; pipecolic acid acts as a neuromodulator for GABAergic neurons, glutamate can be converted to AKG using NAD^+^ and is also a precursor of GABA (Fig. 5).

### *In vitro* and *in vivo* quantitation and validation of pipecolic acid as a potential diagnostic marker of malaria

**Figure 6:**
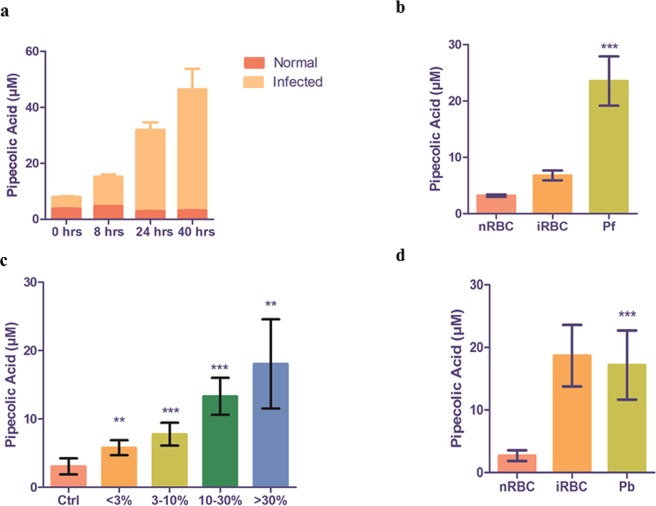
*In vitro* and *in vivo* validation of pipecolic acid as a diagnostic marker for malaria. (**a**) The levels of pipecolic acid increase with time in spent culture medium (Med) of *P*. *falciparum*-infected RBC. At 0 h, medium from both non-infected RBC (nMed) and infected RBC (iMed) show similar levels of pipecolic acid (P = ns, n = 3). A small change was observed between nMed and iMed at 8 h (P = 0.0172, n = 4). At 24 h, a 10-fold change was seen between nMed and iMed (P = 0.0101, n = 4), respectively. At 40 h a ~15-fold increase was observed between nMed and iMed (P = 0.0322, n = 3), respectively. (**b**) Pipecolic acid was measured in nRBC, iRBC and Pf at 40 hrs. A significant 2-fold increase was observed in iRBC compared to nRBC (P- value = 0.0059, n = 4). Importantly, pipecolic acid was present in *P*. *falciparum* lysate (n = 12). (**c**) Pipecolic acid was measured in the plasma of *P*. *berghei* infected mice (n = 4 each set). Mice were divided in 5 groups. Pipecolic acid increased with parasite load; Compared with control mice, those with parasitemia <3%, 3–10%, 10–30% and >30%, the observed fold change in pipecolic acid was 2-fold, 2.5-fold, 4-fold and 6-fold, respectively (P = 0.0002, n = 4). (**d**) Pipecolic acid was measured in nRBC, iRBC from mice with <3% and 3–10% parasitemia and Pb lysate. A significant 6-fold increase was observed in iRBC compared to nRBC (P- value < 0.0001, n = 4). Importantly, pipecolic acid was present in *P*. *berghei* lysate. nMed and nRBC represent the supernatant and saponin lysate of normal RBCs, respectively. iMed and iRBC represent the supernatant and saponin lysate of infected RBCs at the mentioned parasitemias, respectively. Pf and Pb correspond to *P*. *falciparum* and *P*. *berghei*, respectively.

Targeted metabolomics was used to quantify levels of pipecolic acid from *P*. *falciparum-*infected culture supernatant at 0 h, 8 h, 24 h and 40 h post invasion (Fig. 6a). Differences between pipecolic acid levels first became evident at 8 h and by 24 h differences pipecolic acid were more pronounced (2.894 ± 0.069 µM vs 29.08 ± 2.646 µ M in test; P = 0.0101, n = 3). At 40 h the level of extracellular pipecolic acid from uninfected red blood cells was essentially unchanged (3.119 ± 0.1281 µM), but was markedly increased in the supernatant of schizont-infected erythrocytes (43.27 ± 3.82 µM; P = 0.0322, n = 3). The intracellular levels of pipecolic acid were measured following saponin lysis of RBC. As shown in Fig. 6b, at 5% parasitemia, RBC lysate of infected culture had 2-fold higher pipecolic acid levels than corresponding normal RBC lysate (6.789 ± 0.877 µM vs 3.201 ± 0.2231 µM; P- value = 0.0059, n = 4).

Pipecolic acid was found to be present in plasma of *P*. *berghei*-infected mice. Five sets of mice (n = 4 in each) were used which corresponded to control mice, mice with parasitemia of <3%, 3–10%, 10–30% and >30%, respectively. Even at low parasitemia of <3%, mice exhibited measurable and significant hyperpipecolatemia (5.796 ± 0.4900 µM vs 3.066 ± 0.5265 µM in control; P value = 0.0053). With increase in parasitemia, there was a progressive increase in levels of pipecolic acid (7.785 ± 0.5844 µM, 13.31 ± 1.353 µM, 18.05 ± 3.257 µM in mice with parasitemia 3–10%, 10–30% and >30%, respectively (Fig. 6c). Thus, the concentration of pipecolic acid in the plasma of *P*. *berghei*-infected mice is directly proportional to the parasite load (Fig. 6c). Further, following saponin lysis, RBC lysate was collected from control mice and mice with parasitemia <3% and 3–10%. Pipecolic acid was ~2.5-fold increased in parasitized mice as compared to control mice RBC lysate (P < 0.0001). Interestingly, the parasite lysate also had high levels of pipecolic acid (17.18 ± 2.253 µM; n = 6) thus, confirming the parasite-origin of the metabolite (Fig. 6d).

## Discussion

Metabolic pathways of obligate, intracellular pathogens are highly intertwined with their host’s to support pathogen growth, whilst the infected host struggles to maintain overall homeostasis. Here, we’ve shown that intra-erythrocyte parasite development perturbed metabolic pathways involved in lipid, amino acid, carbohydrate and vitamin metabolism. Of the 141 metabolites analyzed, only 10 were common to ring-, trophozoite- and schizont-infected erythrocytes, highlighting the metabolic heterogeneity between the different developmental stages. We observed alterations in lipid, amino acid and carbohydrate metabolism pathways, as also reported by recent studies on *Plasmodium* metabolomics^2,9,11,29,30^. Previous studies have described periodic fluctuations in gene expression and metabolite profiles during the 48 h intra-RBC cycle^2^, but we deliberately stopped our analysis at 40 h to avoid red blood cell lysis linked to merozoite egress. Four metabolites were uniquely and abundantly altered in *P*. *falciparum*-infected red blood cells; namely, pipecolic acid, GABA, alpha ketoglutarate and NAD (Fig. 4).

Previously, pipecolic acid had been detected in serum and urine of *P*. *berghei* and *P*. *vivax*-infected hosts, but was thought to stem from the metabolism of gut microbiota^11,29,30^. We performed targeted metabolomics to quantify pipecolic acid both *in vitro* and *in vivo* in plasma of *P*. *berghei*-infected mice. Pipecolic acid levels were maximal released into the culture supernatant by *P*. *falciparum* schizont-infected erythrocytes, and *in vivo*, pipecolic acid levels were uniquely detected in plasma of *P*. *berghei*-infected mice and increased with parasite load. As hyperpipecolatemia is seen in patients with Zellweger’s syndrome^36^ it argues that released pipecolic acid could contribute to malaria pathogenesis. In addition, pipecolic acid can play a regulatory role in GABA-ergic neurotransmission acting as a neuromodulator^44^ and is known to induce oxidative stress *in vitro* in cerebral cortex of experimental rats^45^. Moreover, the redox metabolite homocysteine can compromise the blood brain barrier integrity and be toxic to the nervous system^25,26^. Significant changes in the levels of homocysteine, GABA and pipecolic acid argues that if these metabolites are released extracellularly by *P*. *falciparum* schizont-infected red blood cells either before a breach in the blood brain barrier, or after parasites are sequestered in the brain, their release could provoke some of the neurological symptoms associated with cerebral malaria (Fig. 5).

*P*. *yoelli*-infected erythrocytes have been shown to release ATP that signals via P2Y receptors^17^. However, our study did not detect ATP, but AMP, likely due to rapid conversion of ATP to AMP via the ecto-5′-nuclotidase CD39 and indeed, in a pilot study using 2 groups of 3 mice showed that inhibiting CD39 reduced growth of *P*. *berghei* by 40% over a 10-day period (not shown). The greater effect on parasite growth following inhibition of CD73 suggests that conversion of AMP to adenosine contributes more to parasite growth^16^. This provides an example of how study of global metabolomics can enable us to understand *in vivo* relevance of inhibition by certain molecules.There’s substantial evidence that metabolomic analyses can significantly deepen our understanding about the parasite’s metabolism, response to different drug interventions and physiologically relevant perturbations of its environment. It is also clear that metabolic plasticity and pathways of *Plasmodium* sp varies vastly from free-living protozoans and higher eukaryotes^46^. In the future, this knowledge can help identify parasite-induced alterations in the host. MultiOMICS approaches unifying genomics, transcriptomics, proteomics and metabolomics combined has the potential to improve diagnosis and treatment of malaria.

## Experimental Procedures

### Cell culturing

#### Plasmodium falciparum

*P*. *falciparum* 3D7A was cultured in human O+ erythrocytes in complete RPMI 1640 (Sigma Aldrich) medium supplemented with 0.5% (w/v) Albumax II (Invitrogen), 0.2% (w/v) NaHCO3, 0.2% (w/v) Glucose, 200 µM Hypoxanthine and 5 µg/L Gentamycin. Fresh media was added in every 24 h interval. Cultures were split once the parasitemia reached 5% and were supplemented with fresh RBCs. O+ whole blood was obtained from Red Cross Blood Bank Society, Bangalore. Saponin lysis was performed as described elsewhere^1^.

#### Plasmodium berghei

Mice were maintained, and experiments were performed as per the principles, guidelines and methods approved by the Institutional Animal Ethics Committee (IAEC) of the Indian Institute of Science, Bangalore in accordance with Indian National Law on animal care and use (IAEC Ethical approval reference number: CAF/Ethics/269/2012). *P*. *berghei* ANKA strain was obtained from MR4 and maintained by successive intra-peritoneal injection (10^5^ infected erythrocytes; 200 µl) in Swiss female mice (6–8 weeks old; 22–25 g). Blood collected from tail vein was used to monitor parasitemia using Giemsa stained smears post 48 h of infection and was scored microscopically. Mice were considered uninfected if no parasites were observed in 50-fields of view.

### Global Metabolic Profiling of culture supernatant

Global biochemical profiles were determined in human red blood cells (control) and *Plasmodium falciparum*-infected red blood cell culture (test) media supernatants collected at 8 h, 24 h and 40 h corresponding to three stages of RBC infection; ring (8 h), trophozoites (24 h) and schizonts (40 h). Sample preparations and GC/MS and LC/MS/MS were performed in Metabolon. Detailed methods are discussed in Supplementary File 1.

### Preparing samples for measuring pipecolic acid in the culture supernatant

Equal number of normal RBCs and parasitized RBCs (parasitemia ranging between 3–4%) were incubated at 5% haematocrit as per previous conditions. After 0, 8, 24 and 40 h, the spent medium was processed for LC-MS and was analyzed for pipecolic acid.

### Processing of samples and LC-MS detection of pipecolic acid

To the *in vitro* culture supernatant, 10 v/v 0.1% formic acid (Sigma) in methanol was added to precipitate the proteins and extract the metabolites. For quantification from mice plasma, 5 v/v 0.1% formic acid in methanol was used. Samples were incubated on ice for 10 min. Post incubation, samples were spun at 14,000 g for 10 min. The supernatant obtained was then subjected to HPLC-MS analysis. For detailed methods and MS parameters please refer Supplementary File 2. Quantification of metabolites was carried out using HPLC-MS/MS using Agilent 1200 series HPLC coupled with tandem Agilent 6460 QQQ mass spectrometer.

### Statistical Analysis

Results were reported as Mean ± S.E.M. Grouped data was statistically analyzed using one-way ANOVA. For paired comparisons, two-tailed P-test was used. All analysis was done using GraphPad Prism 5.0.

## Supplementary information

Supplementary Information

Dataset 1
