# Supplementary material for: Insights into physiological roles of unique metabolites released from Plasmodium-infected RBCs and their potential as clinical biomarkers for malaria
Source: Sci Rep. 2019 Feb 27;9:2875. doi: 10.1038/s41598-018-37816-9 (PMC6393545; doi:10.1038/s41598-018-37816-9)
Supplement: Supplementary file 1 — Supplementary Information [file 41598_2018_37816_MOESM1_ESM.pdf]

Insights into physiological roles of unique metabolites released from *Plasmodium*-infected RBCs and their potential as clinical biomarkers for malaria

Divya Beri[1*], Ghania Ramdani[2,3*], Balu Balan[1], Darshak Gadara[1], Mukta Poojary[1], Laurence Momeux[2,3], Utpal Tatu[1,#] and Gordon Langsley[2,3#]

[1]Department of Biochemistry, Indian Institute of Science, Bangalore – 560012, India

[2]Inserm U1016, Cnrs UMR8104, Cochin Institute, Paris, 75014 France.

[3]Laboratoire de Biologie Cellulaire Comparative des Apicomplexes, Faculté de Médecine, Université Paris Descartes - Sorbonne Paris Cité, France.

*Co-first authors

#Co-corresponding authors

Supplementary Files

Supplementary File 1: Description of detailed methods and statistical analysis adopted for the study.

Sample Preparation:

Thirty-one (five biological replicates of each of the three time points for both control and P. falciparum-infected sample and one medium control) samples were stored at -80˚C. The sample preparation process was carried out using the automated MicroLab STAR® system from Hamilton Company. Recovery standards were added prior to the first step in the extraction process for QC purposes. Sample preparation was conducted using a proprietary series of organic and aqueous extractions to remove the protein fraction while allowing maximum recovery of small molecules. The resulting extract was divided into two fractions; one for analysis by LC and one for analysis by GC. Samples were placed briefly on a TurboVap® (Zymark) to remove the organic solvent. Each sample was then frozen and dried under vacuum. Samples were then prepared for the appropriate instrument, either LC/MS or GC/MS.

Liquid chromatography/Mass Spectrometry (LC/MS, LC/MS^2): The LC/MS portion of the platform was based on a Waters ACQUITY UPLC and a Thermo-Finnigan LTQ mass spectrometer, which consisted of an electrospray ionization (ESI) source and linear ion-trap (LIT) mass analyzer. The sample extract was split into two aliquots, dried, then reconstituted in acidic or basic LC-compatible solvents, each of which contained 11 or more injection standards at fixed concentrations. One aliquot was analyzed using acidic positive ion optimized conditions and the other using basic negative ion optimized conditions in two independent injections using separate dedicated columns. Extracts reconstituted in acidic conditions were gradient eluted using water and methanol both containing 0.1% Formic acid,

while the basic extracts, which also used water/methanol, contained 6.5mM Ammonium Bicarbonate. The MS analysis alternated between MS and data-dependent MS^2 scans using dynamic exclusion.

Gas chromatography/Mass Spectrometry (GC/MS): The samples destined for GC/MS analysis were re-dried under vacuum desiccation for a minimum of 24 hours prior to being derivatized under dried nitrogen using bistrimethyl-silyl-trifluoroacetamide (BSTFA). The GC column was 5% phenyl and the temperature ramp is from 40° to 300° C in a 16 minute period. Samples were analyzed on a Thermo-Finnigan Trace DSQ fast-scanning single-quadrupole mass spectrometer using electron impact ionization. The instrument was tuned and calibrated for mass resolution and mass accuracy on a daily basis. The information output from the raw data files was automatically extracted as discussed below.

Accurate Mass Determination and MS/MS fragmentation (LC/MS), (LC/MS/MS): The LC/MS portion of the platform was based on a Waters ACQUITY UPLC and a Thermo-Finnigan LTQ-FT mass spectrometer, which had a linear ion-trap (LIT) front end and a Fourier transform ion cyclotron resonance (FT-ICR) mass spectrometer backend. For ions with counts greater than 2 million, an accurate mass measurement could be performed. Accurate mass measurements could be made on the parent ion as well as fragments. The typical mass error was less than 5 ppm. Ions with less than two million counts require a greater amount of effort to characterize. Fragmentation spectra (MS/MS) were typically generated in data dependent manner, but if necessary, targeted MS/MS could be employed, such as in the case of lower level signals.

Bioinformatics: The informatics system consisted of four major components, the Laboratory Information Management System (LIMS), the data extraction and peak-identification software, data processing tools for QC and compound identification, and a collection of information interpretation and visualization tools for use by data analysts. The hardware and software foundations for these informatics components were the LAN backbone, and a database server running Oracle 10.2.0.1 Enterprise Edition.

LIMS: The purpose of the Metabolon LIMS system was to enable fully auditable laboratory automation through a secure, easy to use, and highly specialized system. The scope of the Metabolon LIMS system encompasses sample accessioning, sample preparation and instrumental analysis and reporting and advanced data analysis. All of the subsequent software systems are grounded in the LIMS data structures. It has been modified to leverage and interface with the in-house information extraction and data visualization systems, as well as third party instrumentation and data analysis software.

Data Extraction and Quality Assurance: The data extraction of the raw mass spec data files yielded information that could loaded into a relational database and manipulated without resorting to BLOB manipulation. Once in the database the information was examined, and appropriate QC limits were imposed. Peaks were identified using Metabolon's proprietary peak integration software, and component parts were stored in a separate and specifically designed complex data structure.

Compound identification: Compounds were identified by comparison to library entries of purified standards or recurrent unknown entities. Identification of known chemical entities was based on comparison to metabolomic library entries of purified standards. As of this writing, more than 1000 commercially available purified standard compounds had been acquired registered into LIMS for distribution to both the LC and GC platforms for

determination of their analytical characteristics. The combination of chromatographic properties and mass spectra gave an indication of a match to the specific compound or an isobaric entity. Additional entities could be identified by virtue of their recurrent nature (both chromatographic and mass spectral). These compounds have the potential to be identified by future acquisition of a matching purified standard or by classical structural analysis.

Curation: A variety of curation procedures were carried out to ensure that a high-quality data set was made available for statistical analysis and data interpretation. The QC and curation processes were designed to ensure accurate and consistent identification of true chemical entities, and to remove those representing system artifacts, mis-assignments, and background noise.

Metabolon data analysts use proprietary visualization and interpretation software to confirm the consistency of peak identification among the various samples. Library matches for each compound were checked for each sample and corrected if necessary.

Statistical Calculation: For many studies, two types of statistical analysis are usually performed: (1) significance tests and (2) classification analysis. (1) For pair-wise comparisons we typically perform Welch's t-tests and/or Wilcoxon's rank sum tests. For other statistical designs we may perform various ANOVA procedures (e.g., repeated measures ANOVA). (2) For classification we mainly use random forest analyses. Random forests give an estimate of how well we can classify *individuals* in a *new* data set into each group, in contrast to a t-test, which tests whether the unknown means for two populations are different or not. Random forests create a set of classification trees based on continual sampling of the experimental units and compounds. Then each observation is classified based on the majority votes from all the classification trees. Statistical analyses are performed with the program "R"

Data Quality: Instrument and Process Variability

Instrument variability was determined by calculating the median relative standard deviation (RSD) for the internal standards that were added to each sample prior to injection into the mass spectrometers. Overall process variability was determined by calculating the median RSD for all endogenous metabolites (i.e., non-instrument standards) present in 100% of the Client Matrix samples, which are technical replicates of pooled client samples. Values for instrument and process variability meet Metabolon's acceptance criteria as shown in the table 1

Table 1

QC Sample	***Measurement***	***Median RSD***
Internal Standards	Instrument Variability	7 %
Endogenous Biochemicals	Total Process Variability	22 %

Supplementary File 2: Description of LC-MS/MS parameters for quantification of pipecolic acid using targeted metabolomics.

S2a: HPLC and MS/MS Parameters

	**Pipecolic Acid: (130.1>84.1)**
Column:	Zorbax Eclipse Plus C18 (4.6x 250 mm; 5 micron particle size)
Mobile phase:	Methanol: water 0.3% formic acid (40:60) v/v
Flow rate:	1ml/min
Runtime:	5 mins
Retention time:	2.67 ± 0.3 mins
Extraction:	100 µl sample + 900 µl 10% TCA
Linearity range (ng/mL):	5, 10, 20, 50, 100, 200, 500, 1000, 2000

S2b: Calibration Curve:

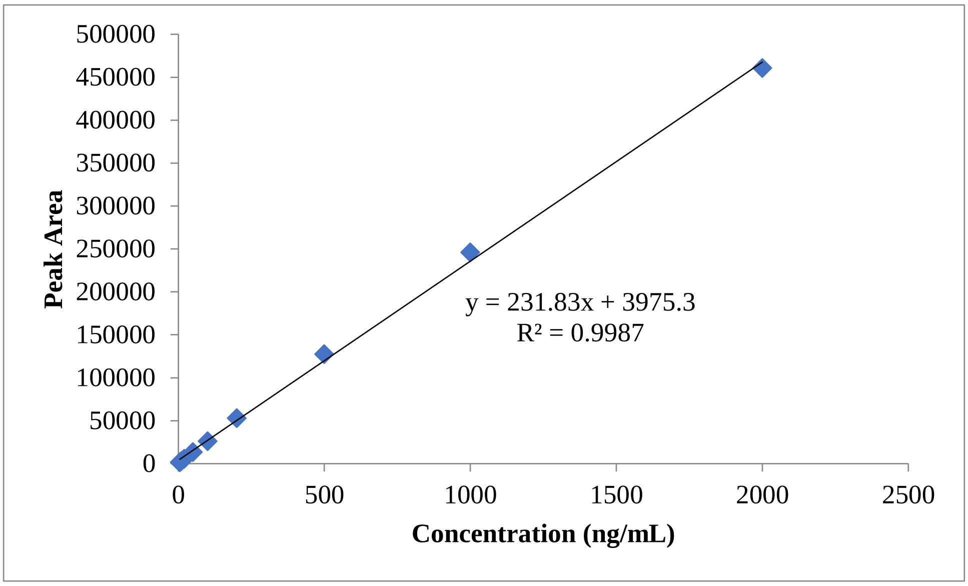

S2c: Ion Chromatogram

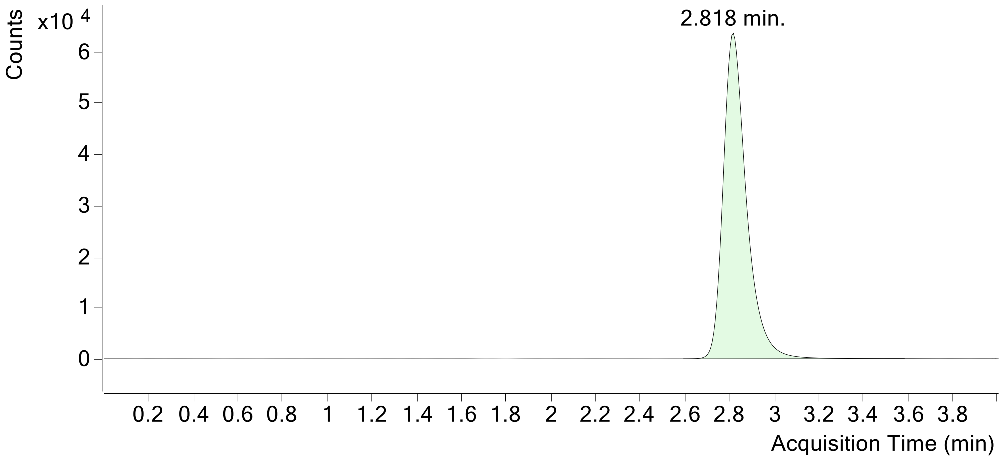

Supplementary Figures and Legends

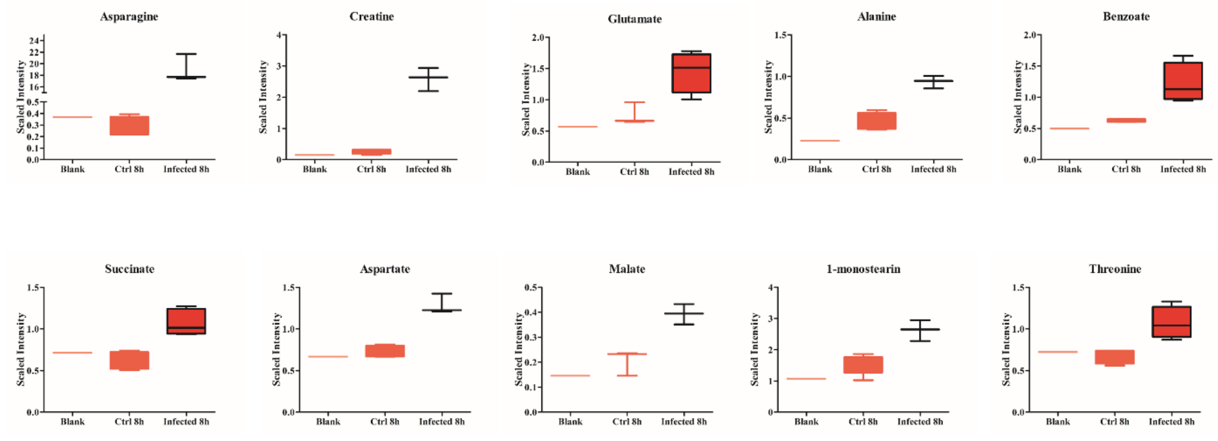

Figure S1a: Top ten metabolites upregulated in the culture supernatant of ring-infected RBC. Y-Axis of the box plots represents scaled intensity and X-Axis indicates the treatment group. **(a)** Box plots depicting the increase in levels of ten metabolites (fold change >1) with maximum fold change among all metabolites measured and p-value<0.05. Most affected metabolites belong to amino acid metabolism thus signifying that it represents the earliest affected pathways following *P. falciparum*-infection of RBC.

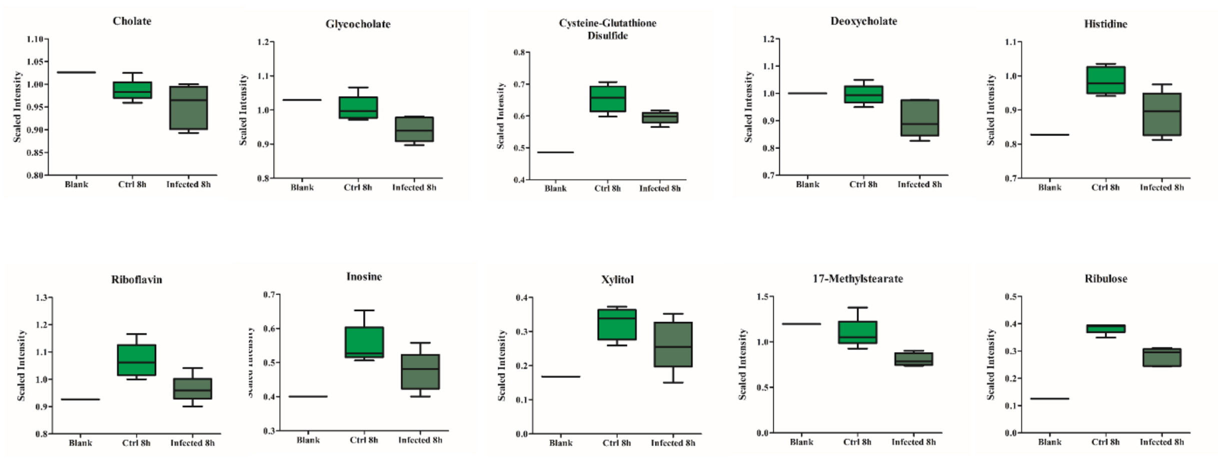

Figure S1b: Top ten metabolites downregulated in the culture supernatant of ring-infected RBC. (**a**) Y-Axis of the box plots represents scaled intensity and X-Axis indicates the treatment group. **(b)** Box plots depicting the decrease in level of ten metabolites with maximum fold change (fold change < 1) and P-value < 0.05. These metabolites include three metabolites related to bile acid metabolism.

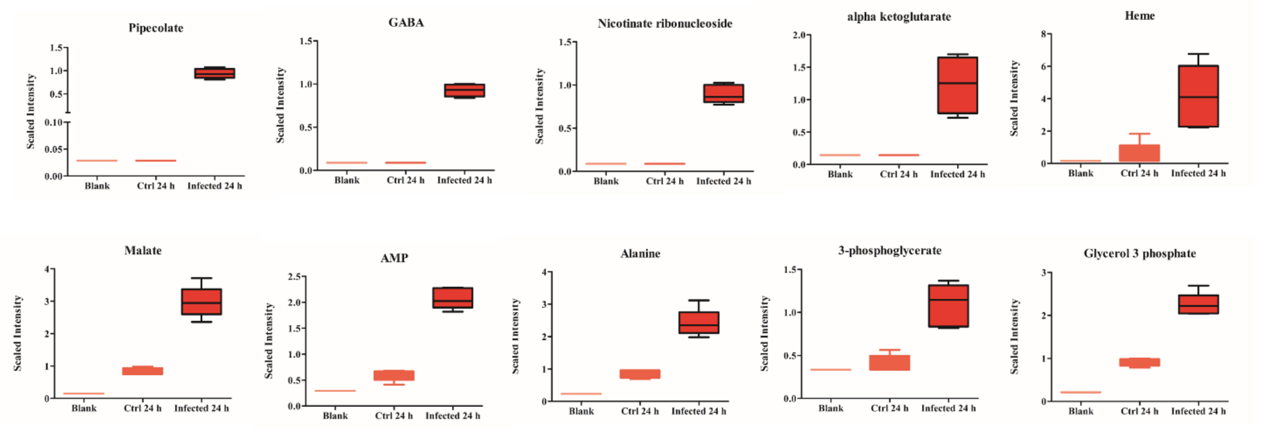

Figure S2a: Top ten metabolites upregulated in the culture supernatant of trophozoite-infected RBC. Y-Axis of the box plots represents scaled intensity and X-Axis indicates the treatment group. **(a)** Box plots depicting the increase in levels of ten maximum fold-change metabolites (fold change > 1) with P-value < 0.05. Amino acid metabolism pathways dominate this list with maximum upregulation seen in pipecolate and GABA. Glucose metabolism related metabolites like alpha ketoglutarate and 3-phosphoglyverate also show pronounced increase. Interestingly, AMP levels also show significant increase at the 24- hour time point.

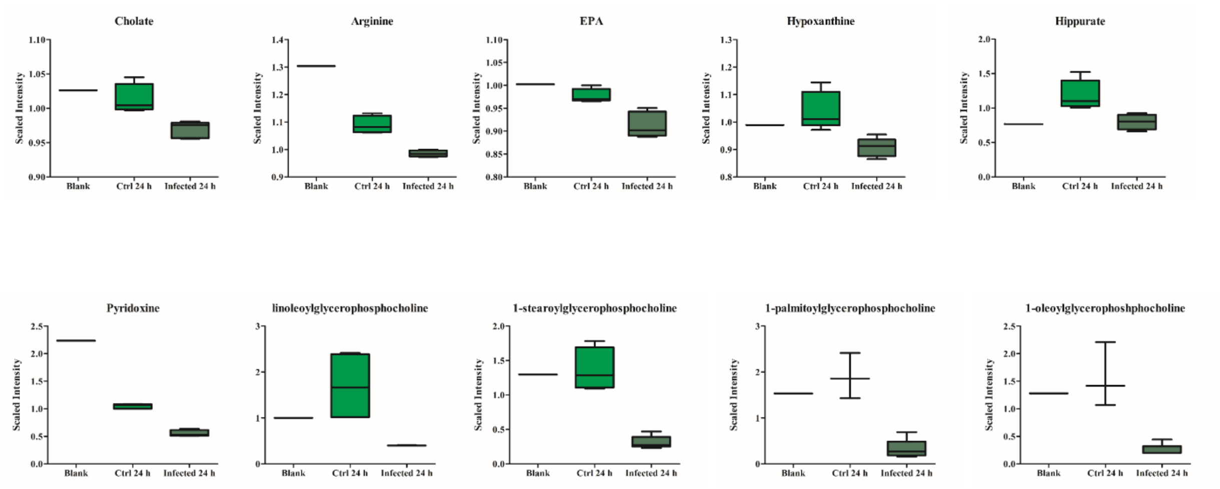

Figure S2b: Top ten metabolites downregulated in the culture supernatant of trophozoite-infected RBC. Y-Axis of the box plots represents scaled intensity and X-Axis indicates the treatment group. **(b)** Box plots depicting the decrease in levels of ten maximum fold-change metabolites (fold change < 1) with p value< 0.05. metabolites related to the cell membrane architecture dominate this group suggesting a change in membrane fatty acids dynamics at this stage of the infection.

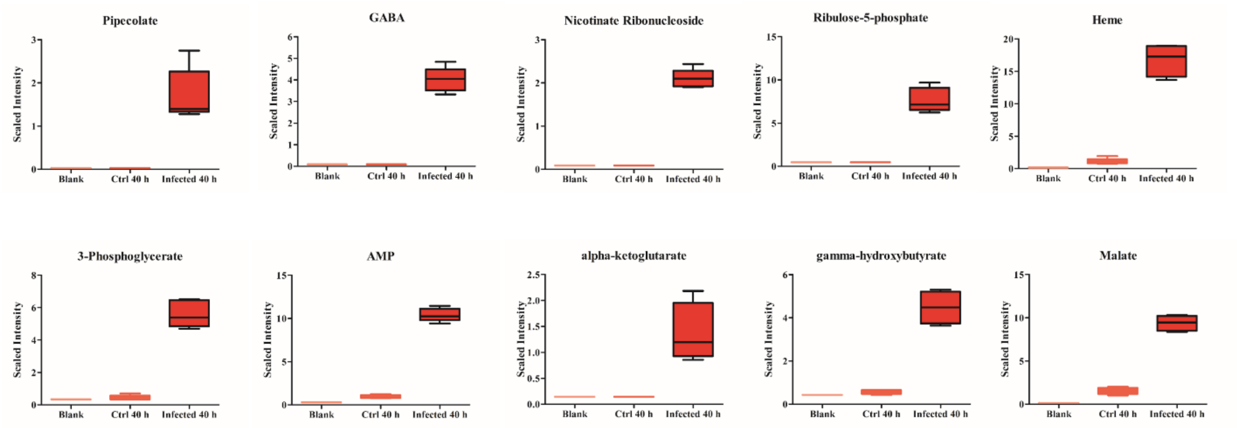

Figure S3a: Top ten metabolites upregulated in the culture supernatant of schizont-infected RBC. Y-Axis of the box plots represents scaled intensity and X-Axis indicates the treatment group. **(a)** Box plots depicting the increase in levels of ten metabolites exhibiting maximum fold change (fold change > 1) with p-value < 0.05. Pipecolate, GABA, nicotinate ribonucleoside are the top metabolites affected at this stage. Also, metabolites like AMP, 3-phosphoglycerate and heme show a pronounced increase suggesting a dramatic change in parasite and/or host metabolism at this stage.

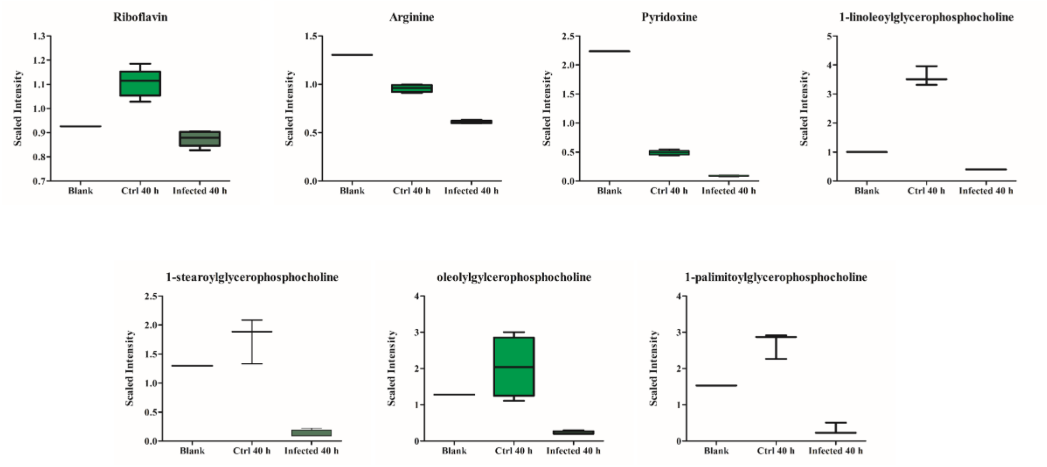

Figure S3b: Top ten metabolites downregulated in the culture supernatant of schizont-infected RBC. Y-Axis of the box plots represents scaled intensity and X-Axis indicates the treatment group. (**b**) Box plots depicting the top ten metabolites that show maximum downregulation (fold change < 1) and p-value < 0.05. Cell membrane related metabolites dominate this group closely followed by critical vitamins riboflavin and pyridoxine.
